# Soybean yield, nutrient uptake and stoichiometry under different climate regions of northeast China

**DOI:** 10.1038/s41598-020-65447-6

**Published:** 2020-05-21

**Authors:** Shicheng Zhao, Xinpeng Xu, Dan Wei, Xiaomao Lin, Shaojun Qiu, Ignacio Ciampitti, Ping He

**Affiliations:** 1 Ministry of Agriculture Key Laboratory of Plant Nutrition and Fertilizer, Institute of Agricultural Resources and Regional Planning, Chinese Academy of Agricultural Sciences, Beijing, 100081 P.R. China; 2 Institute of Plant Nutrition and Resources, Beijing Academy of Agricultural and Forestry Sciences, Beijing, 100097 P.R. China; 3 Department of Agronomy, Kansas State University, Manhattan, KS 66506 USA

**Keywords:** Fertilization, Governance

## Abstract

Climate and soil fertility influence seed yield, nutrient uptake, and nutrient stoichiometry in the plant. We collected soybean [*Glycine max* (L.) Merr.] data were collected from field experiments in northeast China (warm and cold regions) to study the effect of temperature variations during the crop growing season on seed yield, nutrient uptake and stoichiometry from 2001 to 2017. Soybean seed yield has been increased in the cold region but not in the warm region, where average seed yield was higher. The indigenous nitrogen (N) supply followed the same trend as yield, greater in warmer environments but also increasing over time. The internal efficiency (IE) of N and potassium (K) performed similarly in both climate regions, but phosphorus (P) IE was 30% greater in the warm region than the cold region. For soybean nutrient uptake ratio, the N/K ratio was similar between both regions; however, the N/P ratio was greater in the warmer region relative to the colder region. Overall, the higher temperature experienced in the warm region increased soybean seed yield relative to the cold region, and high soil P accumulation caused soybean P luxury uptake in the cold region of northeast China.

## Introduction

Soybean (*Glycine max* [L.] Merr) is an important dual-purpose crop grown worldwide as source of both vegetable protein and oil. Because of its function of biological nitrogen fixation (BNF), soybean is also an important crop in rotational cropping systems designed for intensive production^1^. Global soybean production was approximately 306 million Mg in 2016^2^, but still inadequate for meeting the increasing global demand^3^. The main factors limiting soybean yields at the farm-scale are related to the environment, genetics, crop management, and their interactions^4^.

The imbalance between nutrients input from fertilization and nutrient demand of soybean is a critical factor constraining soybean seed yields^5^. Soybean has a particularly high nutrient requirement, especially for N due to its high seed protein content^6,7^. Farmers generally disregard application of N fertilizer because soybean can fix N from the atmosphere in some scenarios; however, BNF cannot meet plant N demand, more specifically under high yield environments or when N fixation is impaired^8^. Because other nutrients, phosphorus (P) for instance, play a critical role in promoting the BNF process, N should be in balance with other macronutrients such as phosphorus (P) and potassium (K) in order to maximize seed yield^8–10^.

Climate conditions generally influence crop phenology, plant physiological functions, soil nutrient supply and final crop yields; thus, impacting the management strategies implemented by farmers for improving yields, and ultimately influencing crop yield^4,11,12^. Lobell and Field^13^ reported that maize (*Zea mays* L.) yield decreased by 17% for each 1 °C increase in mean temperature in the US Corn Belt region. Zhang *et al*.^14^ reported that warming advanced flowering stage, shortened the growth period, and decreased soybean yield by 45% in the north China Plain. As one of management strategies for global warming, Yang *et al*.^11^ selected soybean varieties with a longer growth period to increase seed yield in northeast China.

Balanced nutrition is crucial for attaining high crop yields. The relationship between crop yield and nutrient uptake, such as nutrient internal efficiency (IE) and plant nutrient ratio (e.g. N/P and N/K), is widely used to assess nutrient limitations in crop fields^15–17^. Xu *et al*.^15^ found that maize P uptake was luxury in most of the fields in China based on the P IE both directly calculated and simulated using QUEFTS model. Comparing changes in P in different countries around the globe, MacDonald *et al*.^18^ reported that the largest P surpluses were spatially clustered in the U.S. and South Asia, while the largest P deficits were concentrated in South America and Eastern Europe.

China is one of main soybean producing and consuming countries in the world, and the total planting area and seed yield of soybean were 8.2 × 10^6^ ha and 1.53 × 10^7^ Mg in China in 2018, respectively^19^. The negative balance between production and consumption is compensated by importing about 8–10 × 10^7^ Mg year^−1^ of soybean from other countries^20^. The northeast China is a main soybean production area, accounting for 60–70% of total soybean planting area and seed yield in China^21^. Wang *et al*.^12^ reported the southern region of northeast China was most favorable for crop growth due to its higher temperature and conducive climate resources. Until recently, very few studies have been conducted to assess the effect of temperature on soybean yield, nutrient uptake and stoichiometry. Therefore, our objectives in this study was to assess the effect of varied temperature on soybean seed yield, nutrient uptake, and stoichiometry in northeast China.

## Results

### Average daily temperature and total precipitation during soybean growing season

**Figure 1:**
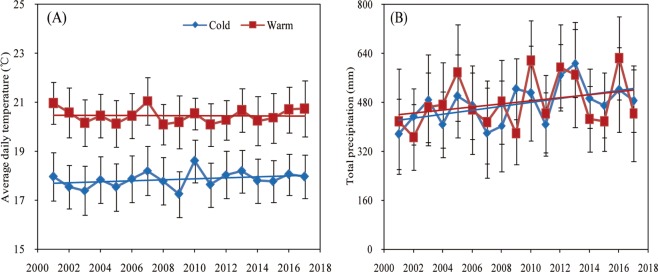
Change in average daily temperature (panel A) and total precipitation (panel B) during the soybean growing season (May to September) in two climate regions (herein termed as cold and warm) of northeast China (2001–2017). Error bars indicate the standard deviation.

Average daily temperature during the soybean growing period did not change significantly in the warm region, but it increased by 0.29 °C in the cold region from 2001–2009 to 2010–2017 (Fig. 1). Average total precipitation during the soybean growing season was similar in both regions and increased at a similar rate over time (Table 1). In May, the warmer region presented greater average temperature and accumulated growing days degree, but lower total precipitation relative to the colder region.

**Table 1:** Average daily air temperature and total precipitation during the soybean growing season (from May to September) and in May under cold and warm regions of northeast China (2001–2017).

Regio	Average daily temperature from May to Sept (°C)	Average daily maximum temperature from May to Sept (°C)	Average total precipitation from May to Sept (mm)	Average daily temperature in May (°C)	Average total precipitation in May (mm)
Cold	17.9	25.6	477.3	14.2	62.4
Warm	20.6	27.4	473.7	17.0	54.3

September, Step.

### Soybean seed yield

**Figure 2:**
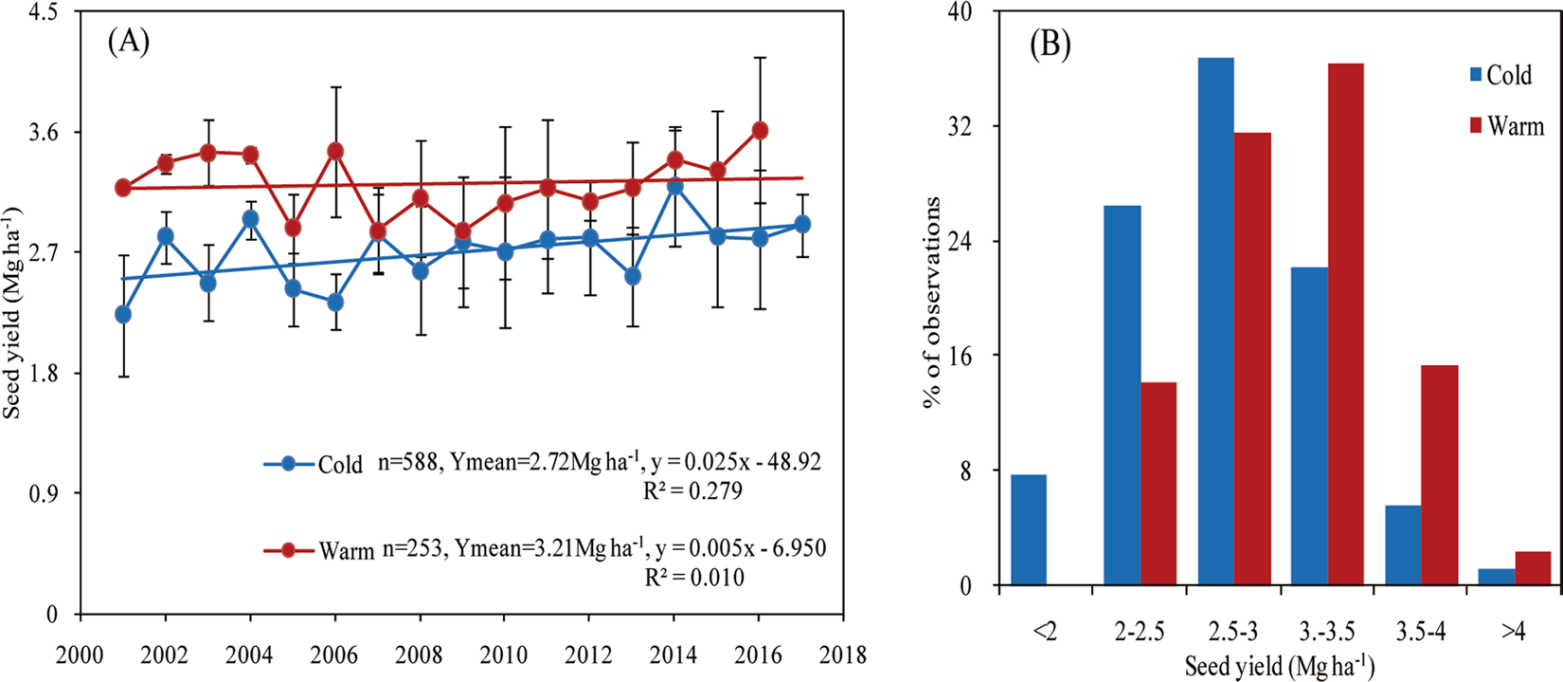
Soybean seed yield in two climate regions (herein termed as cold and warm) of northeast China (2001–2017), yield data obtained from optimum nutrient treatment (panel A), and frequency (in percentage) of observations for each seed yield category (panel B). For panel A, error bars indicate the standard deviation.

Soybean seed yield did not significantly change in the warm region, but experienced an increasing trend over-time in the cold region from 2001 to 2017. Overall, average seed yield was greater in the warmer relative to the colder regions (Fig. 2). A greater proportion of yield observations (68%) within the warmer region presented an overall broader yield range (from 2.5 to 3.5 Mg ha^−1^), but with similar variation relative to the colder region, with 63% of the yield observations ranged from 2 to 3 Mg ha^−1^.

### Soil nutrient partial factor productivity and indigenous nutrient supply

**Figure 3:**
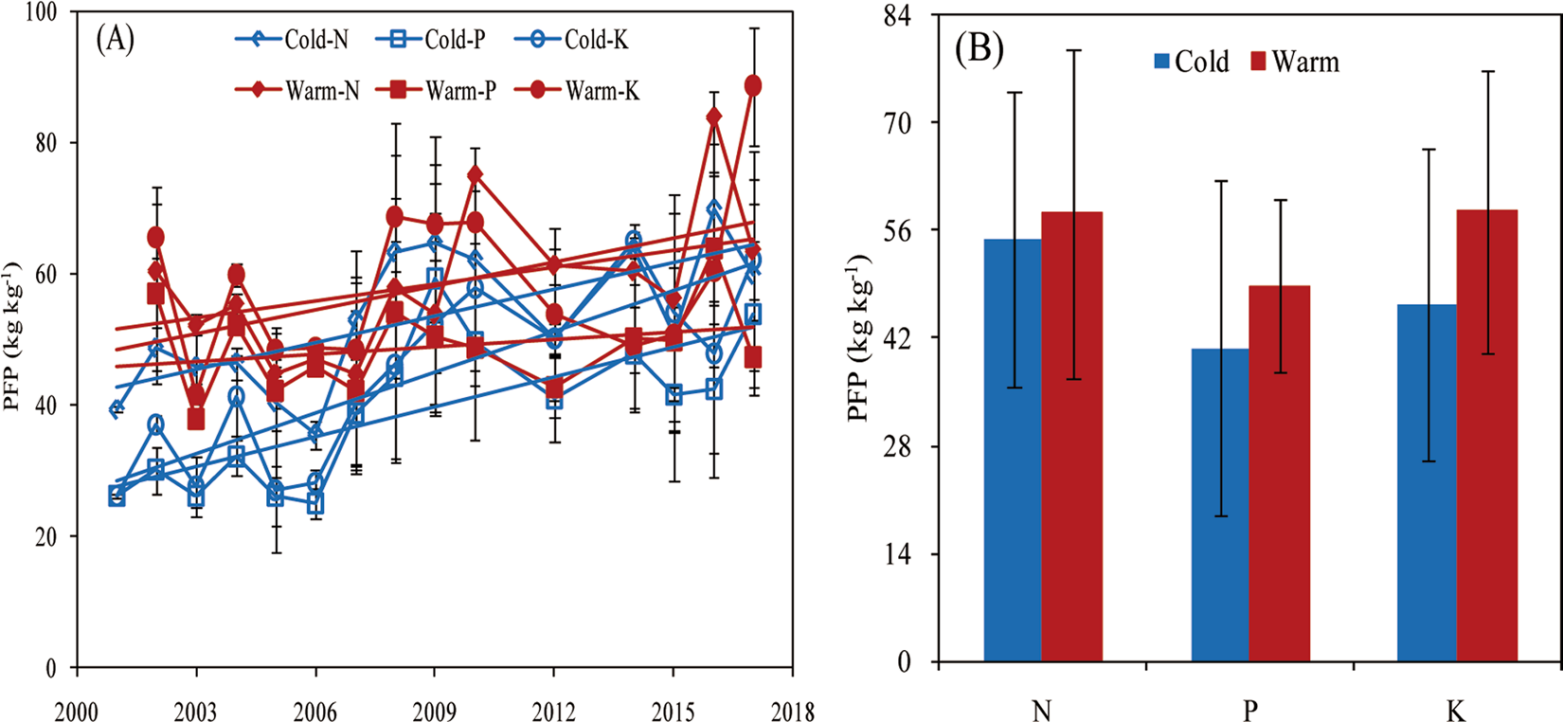
Change in nutrient partial factor productivity (PFP) over time (panel A) and average PFP of soybean for nitrogen (N), phosphorous (P), and potassium (K) (panel B) across the historical period in two climate regions (herein termed as cold and warm) of northeast China (2001–2017) (Data from optimum nutrient treatment). For both panels, error bars indicate the standard deviation.

**Figure 4:**
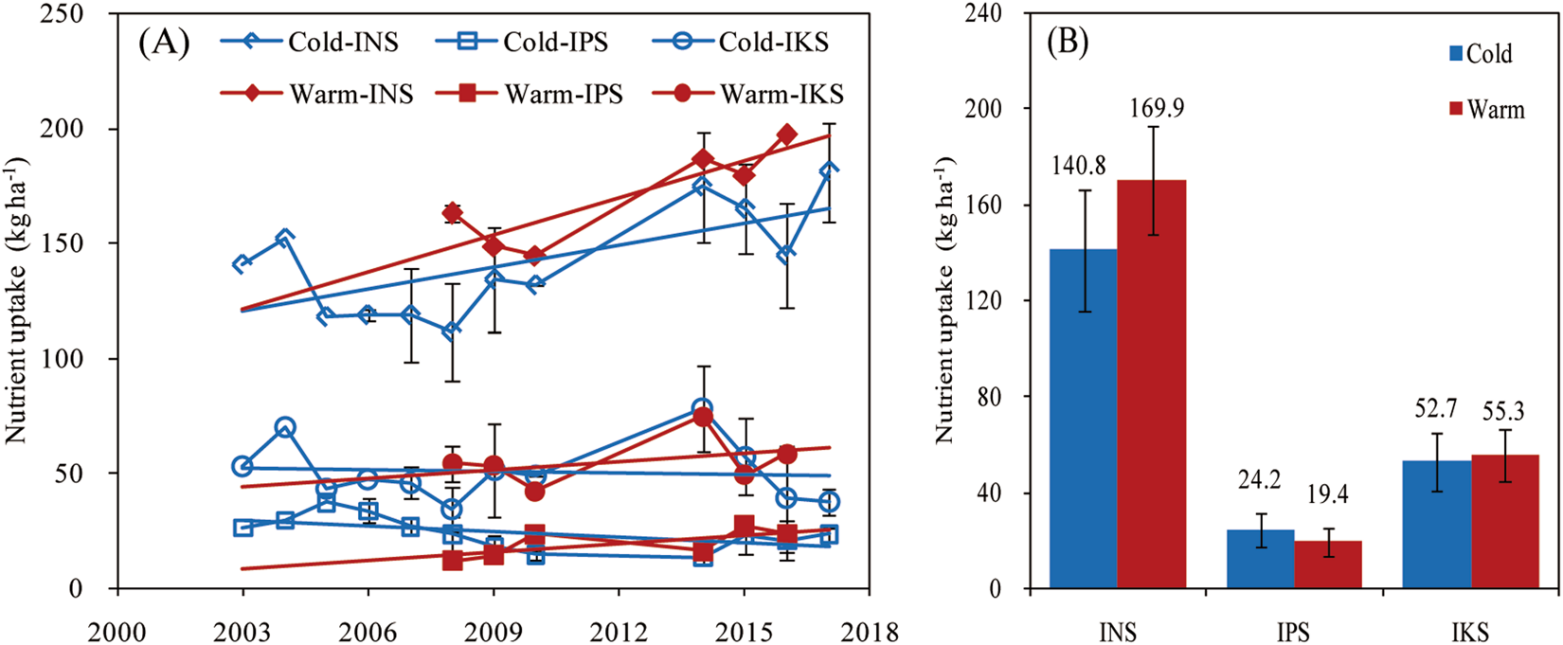
Change in soil indigenous nutrient supply for nitrogen (INS), phosphorous (IPS), and potassium (IKS) over time (panel A) and average soil indigenous nutrient supply across the historical period (panel B) in two climate regions (herein termed as cold and warm) of northeast China (2001–2017). Error bars indicate the standard deviation.

The nutrients nutrient partial factor productivity (PFP) presented a gradual increase from 2000 to 2017 in both regions, and all nutrients PFP showed an increase trend in warm region compared with that in the cold region (Fig. 3). The indigenous P supply (IPS) and indigenous K supply (IKS) did not change in the cold region from 2001 to 2017, but their supply increased by 5.7 and 10.9 kg ha^−1^ from 2008–2011 to 2014–2016, respectively, for P and K in the warm region, and the indigenous N supply (INS) significantly increased with time in both regions (Fig. 4), but with greater INS in warm relative to cold regions.

### Nutrient internal efficiency

**Figure 5:**
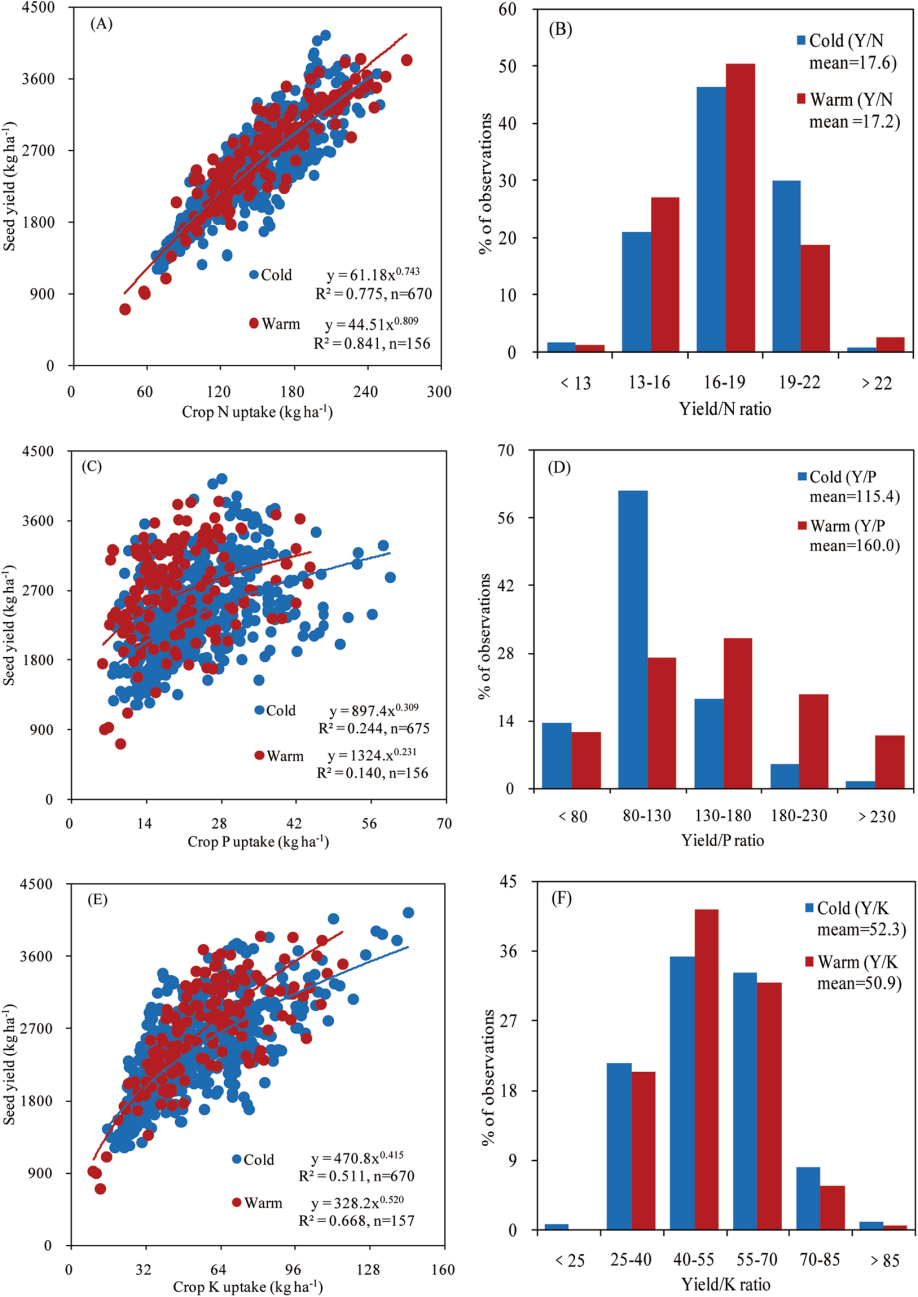
Relationship between soybean seed yield (Y) and total aboveground nutrient uptake (nitrogen, N–panel A, phosphorous, P– panel C, and potassium, K–panel E) and frequency distribution of nutrient internal efficiency data, yield to nutrient uptake ratio, for N (panel B), P (panel D), and K (panel F) in all treatments in two climate regions (herein termed as cold and warm) of northeast China (2001–2017). Trend line fitted for the yield and nutrient uptake in panels A, C, and E have the same color as treatment data. Due to lack of significant difference (p > 0.05) in slope of different treatments, the data of all treatments were pooled in the same region.

Seed yield and aboveground crop N uptake (NIE, slope) fitted the same model for both climate regions (Fig. 5A), with 76% of the yield/N ratio data ranging from 16 to 22 kg seed kg^−1^ N in the cold region, and 77% of the yield/N ratio data ranging from 13 to 19 kg seed kg^−1^ N in the warm region (Fig. 5B). The average yield/N ratio did not differ significantly between two regions. The relationship between seed yield and P uptake differed for the climate regions (Fig. 5C). For the yield/P ratio, 62% of the data ranged from 80 to 130 kg seed kg^−1^ P in the cold region, while 58% of the yield/P ratio ranged from 130 to 180 kg seed kg^−1^ P in the warm region. Overall, the mean yield/P ratio increased by 30% in the warm relative to the cold region (Fig. 5D). The relationship between seed yield and K uptake, the mean yield/K ratio, and the distribution of the N/K ratio all were similar between climate regions (Fig. 5E,F).

### Nutrient stoichiometry

**Figure 6:**
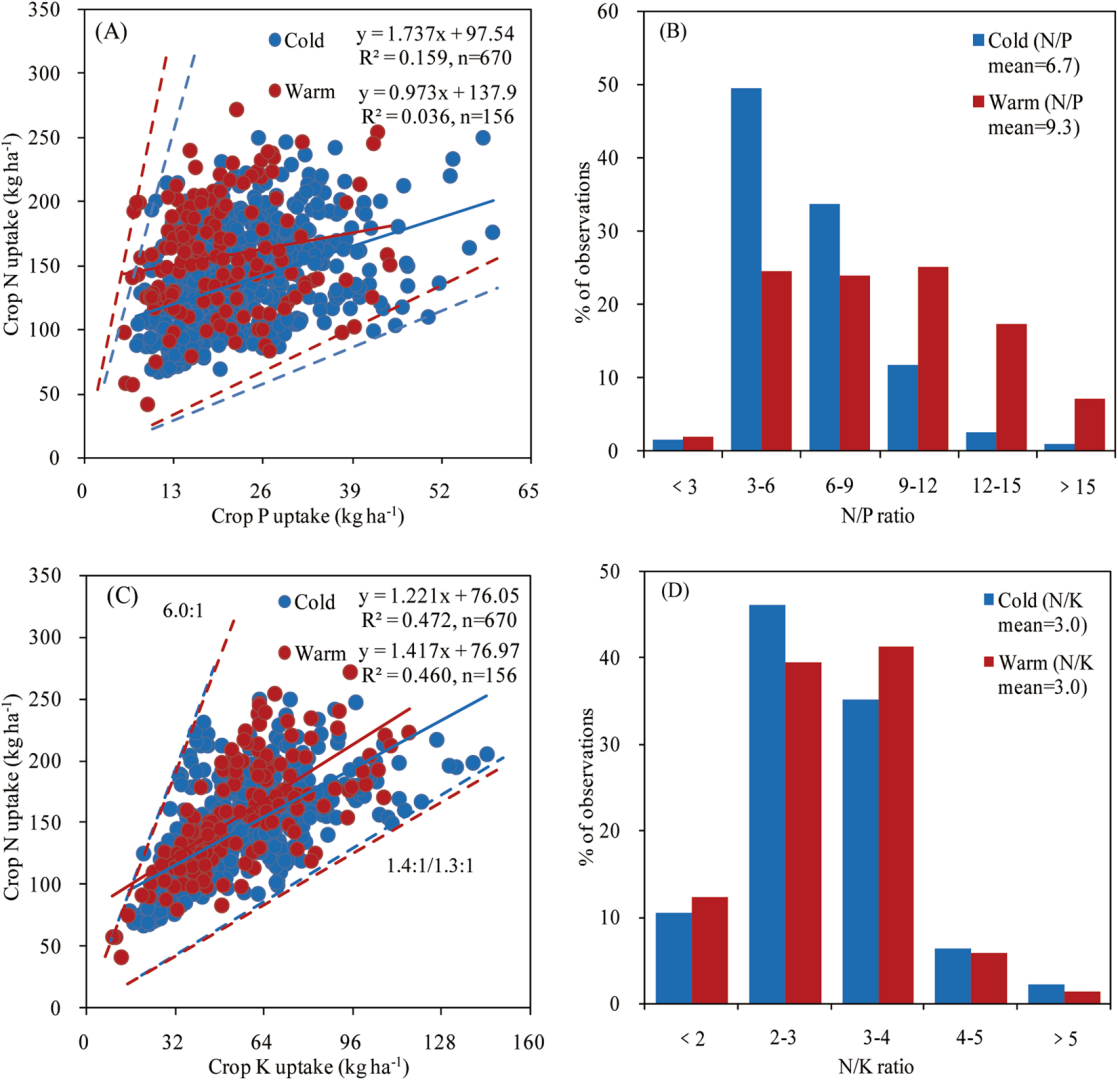
Relationship between soybean total nutrient uptake of nitrogen (N) versus phosphorous (P) uptake (panel A) and N versus potassium (K) uptake (panel C), and data distribution of nutrient ratios for N/P (panel B) and for N/K (panel D) in two climate regions (herein termed as cold and warm) of northeast China (2001–2017). Dotted lines indicate boundaries for maximum and minimum ratio for each dataset.

The N/P ratio ranged from 2.2 to 19.6 in the colder region (averaging 6.7), and ranged from 2.6 to 20.4 for the warmer region (averaging 9.3) (Fig. 6A,B). The fitted slope was greater in the cold compared to the warm region. For the N/P ratio, 49% of data ranged from 3 to 6 in the cold region, while 74% of N/P data ranged from 3 to 12 for the warm region. The N/K ratio was similar and with more than 75% of the data values ranging from 2 to 4 in both regions (Fig. 6C,D).

## Discussion

Our results showed that average daily temperature did not significantly increase over time, especially in the warmer region. Notwithstanding that this result can be inconsistent with the literature in climate warming^22–24^, temperature increases have occured mainly in winter rather than during the crop growing season^25^.

Previous studies reported that a 0.4 °C increase in air temperature advanced soybean anthesis stage by 3.8 days^14^. For each 1 °C increase in mean temperature, growth period was shortened by 7–8, 17, and 7 days for rice, winter wheat, and maize, respectively, and rice yield decreased by 10% or more in portions of east China^26,27^. Our study revealed that soybean seed yield presented a stable or increasing trend in both regions from 2001 to 2017, with the warm region presenting greater seed yield relative to the cold region. The seed yield difference was associated with temperature prevailing in these two regions. Sionit *et al*.^28^ and Pan.^29^ indicated that soybean seed yield increased with increasing daily maximum temperature during seed development in cold regions. Seed filling rate increased with an increase in daily maximum temperature from 18 to 27 °C during seed filling^30^, but seed development was insensitive to the increase in daily maximum temperature between 30 and 35 °C^31^. In this study, the average daily maximum temperature was 25.6 °C (19.9–28.4 °C) and 27.4 °C (26.1–28.8 °C) in the cold and warm regions from 2001 to 2017, respectively. This suggests that the increase in soybean yield may result from the increased seed filling rate under the elevated daily temperature. Higher temperature in the warm region reduced crop failures due to sudden frosts in late summer, created more favorable conditions for soybean growth^11^. The higher potential of photosynthesis-temperature productivity which resulted from higher temperature and solar radiation supplied greater potential productivity for high yielding soybean^12^. Meanwhile, effective agricultural adaptation options are also important to increase soybean seed yield under increasing temperature and growing days degree (GDD). In the warm regions of northeast China, capitalized on temperature and GDD source (Table 2), farmers used to plant soybean cultivars with long growth period and high heat tolerance to increase seed yield^11,32^. In the warm region, the GDD and photo-thermal unit (PTU) accumulated in the emergence, flowering, and physiological maturity stages and the normalized GDD in physiological maturity stage were greater than that in the cold region; however, the normalized GDD were lower in the emergence and flowering stages in the warm region relative to cold region (Table 3). Long sunshine time can supply more light radiation for soybean photosynthesis and growth to increase seed yield^33^. Although slightly longer sunshine time in the cold region relative to the warm region in the same growth duration, higher daily average temperature lead to higher accumulative PTU in different soybean stages of warm region. Ultimately, this indicates that the warm region has higher GDD and PTU from seed filling to maturity stages, which can supply more heat for attaining higher soybean yield, and temperature played more important role in increasing soybean yield compared with sunshine time. Additionally, greater INS also played an important role in increasing seed yield in the warm region. The average soybean seed yield in this study might be overestimated compared with data from the China Agriculture Yearbook^20^, because presented yield data came from optimum fertilization treatment of field experiments, while the unbalanced fertilization is widespread in some areas of northeast China.

**Table 2:** Soil organic matter (OM) and nutrient contents before soybean sowing in the cold and warm regions of northeast China (2001–2017).

Region	OM (g kg^−1^)	TN (g kg^−1^)	TP (g kg^−1^)	TK (g kg^−1^)	AN (mg kg^−1^)	AP (mg kg^−1^)	AK (mg kg^−1^)
Cold	43.4	3.1	0.92	18.7	159.8	35.2	150.8
Warm	23.1	2.2	0.64	—	114.9	28.1	166.0

Total nitrogen, TN; total phosphorus, TP; total potassium, TK; available nitrogen, AN; available phosphorus, AP; available potassium, AK.

**Table 3:** Growing degree days (GDD) and photo thermal unit (PTU) during the soybean growing season (May to September) and emergence, flowering, and physiological maturity, and average soil temperature during the soybean growing season and in May under the cold and warm regions of northeast China (2001–2017).

Region	GDD in whole season (°C)	Average soil Temperature from May to Sept (mm)	Average soil temperature in May (mm)	GDD in emergence (°C) (normalized)	GDD in flowering (°C) (normalized)	GDD in physiological maturity (°C) (normalized)	PTU in emergence (°C)	PTU in Flowering (°C)	PTU in physiological maturity (°C)
Cold	1185	20.9	16.1	37 (0.02)	357 (0.50)	24 (0)	296	2434	218
Warm	1590	23.4	19.3	48 (0)	426 (0.38)	58 (0.01)	460	3052	471

GDD = $\mathop{\sum }\limits_{i=1}^{n}\,(Ti-10\,^\circ {\rm{C}})$, where Ti is the average daily temperature (°C) and the specific baseline temperature of 10 °C was used for soybean^46^, n = 141 and 149 in the cold and warm regions, respectively; PTU = (T − 10 °C) × L, where T is the average daily temperature (°C) and L is sunshine hours^47^. In the cold region, emergence: May 10 to 20, flowing: July 10 to August 10, physiological maturity: September 18 to 28; in the warm region, emergence: May 3 to 13, flowering: July 3 to August 3, physiological maturity: September 18 to 28. The GDDnorm = (X-Xmin)/(Xmax-Xmin).

The increased temperature advanced the date of crop anthesis and maturity, and shorted their growth periods (He *et al*. 2015). But longer sunshine time can extend crop phenological stages, such as photosynthetic time, and increase crop yield (Dong *et al*. 2020).

The N and K IE were similar in both climate regions, but the warm region presented greater P IE than the cold region. Because N concentration in crops is a conservative trait with small variation and a neutral trend over time^3,34,35^. Dobermann^36^ reported that the nutrient IE can indicate the nutrients supply status (deficiency or luxury) under gained yield. In the warm region, greater P IE (and greater yield) compared to the cold region, indicated soybean luxury P uptake occurred in the cold region. Low soil temperature is a main factor limiting soil P supply in northeast China (Table 2), especially in the early stage of soybean or maize crops^37,38^. To meet the P demand for crop growth, farmers often apply more P fertilizer than that crop required, causing an excess and accumulation of P in the cold region of northeast China^39^, potentially causing crop P luxury uptake. The latter is consistent with the results portrayed in this study showing greater IPS and soil P content in the cold region relative to warm region. Although the warm region showed greater INS compared with the cold region, soil N content was lower in the warm region (Table 3), These responses may be due to the fact that higher soybean seed yield and biomass increase N uptake and accumulation.

Nutrient ratios can help predict nutrient limitations for biomass production more effectively than individual nutrient concentrations^3,40,41^. The similar N/K ratio between two climate regions indicated that temperature did not affect the uptake of N and K under different yield levels. Tamagno *et al*.^9^ and Balboa *et al*.^3^ found that mean N/P ratio values in soybean ranged from 10.4, to 13.3 units for Argentina and US. These N/P ratios were greater than our results, potentially related due to differences in soybean varieties and the luxury P uptake in cold region reported in this study the northeast China. The greater N/P ratio in the warmer relative to colder regions, indicated a situation of luxury P uptake, as a consequence of excess of P application to the crop. Therefore, it is necessary to reduce the application of P fertilizer not only to decrease crop production cost and increase P use efficiency in the colder region^42^, but more importantly to reduce the nutrient environmental footprint.

## Conclusions

Our results revealed that varied climate conditions presented great effects on soybean seed yield and nutrient uptake. The warmer region gained greater soybean seed yield than the colder region. Temperature did not influence soybean N and K uptake under different yield levels, but the warm region presented greater P IE relative to the cold region. The cold region presented an excess of soil P due to the untargeted nutrient application relative to the attainable yield, resulting in luxury P uptake. These results indicated that the decrease of P fertilization will be important to increase P use efficiency in the cold region of northeast China.

## Materials and Methods

### Experimental region and data sources

**Figure 7:**
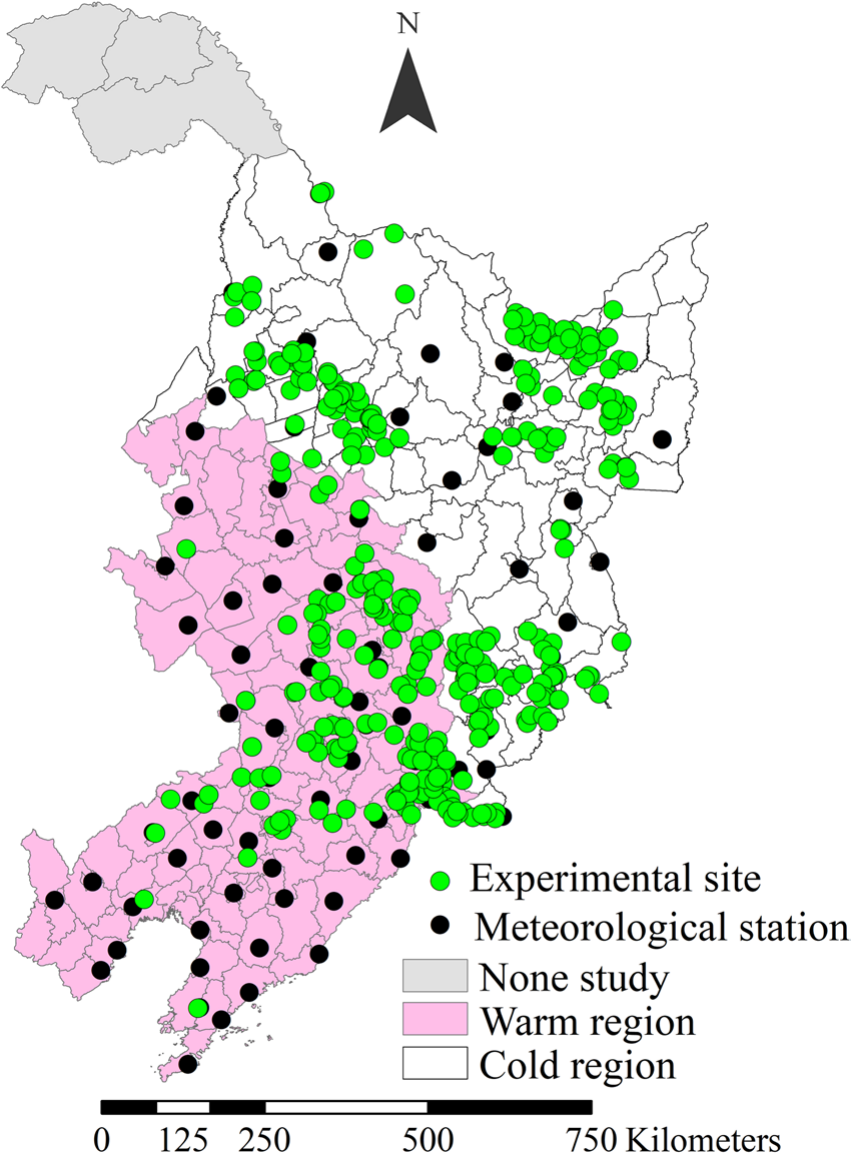
Map of the soybean experimental sites, meteorological stations, and temperature regions in northeast China (2001–2017).

The study area is located in northeast China, and comprised of Heilongjiang, Jilin, and Liaoning Provinces. Soybean was planted in a mono-cropping system in this region (Fig. 7). The database of soybean used in this study consisted of field experiments conducted by the International Plant Nutrition Institute (IPNI) China Program, the Program of Modern Agricultural Industry Technology System for Soybean in China, the National Key Research and Development Program of China, and papers published in scientific journals from 2001 to 2017^43–45^. The experimental treatments in these studies included optimum nutrient combination (N, P, and K were recommended based on soil testing, and the applied rate of N, P_2_O_5_, and K_2_O were 48.1 (30−81), 56.7 (45−90), and 46.5 (31−92) kg ha^−1^, respectively), different fertilizer rates, and nutrient omission treatments (lack of nutrient added) based on optimum nutrient treatment. Seed yield, nutrient uptake in seed and stove, and fertilizer rate were included in these experiments. Soybean varieties used in these experiments were all commonly planted in local production in northeast China. Based on distribution of experimental sites, we selected 81 weather stations operated by the National Meteorological Networks of China Meteorological Administration and collected climatic data (daily average air temperatures, growing degree days, soil temperature, and total precipitation) during the soybean growing period (May to September) from 2001 to 2017 (Fig. 7).

### Data analysis

We analyzed the daily air temperature and total precipitation of these weather stations during the soybean growing season (from May to September) from 2001 to 2017. The average daily air temperature during soybean growing season varied from 16.1 to 22.9 °C across all stations, with the average daily air temperature of 19 °C. Therefore, we divided these experimental sites into warm (>19 °C) and cold (<19 °C) regions based on the average daily temperature, and analyzed soybean data in two climate regions, respectively. Soybean seed yield was adjusted to 135 g kg^−1^ moisture content. The partial factor productivity (PFP, kg ka^−1^) was calculated as the ratio of seed yield to fertilizer rate, and the nutrient internal efficiency as the ratio of seed yield to aboveground nutrient uptake. The indigenous nutrient supply was predicted as the aboveground crop nutrient uptake for each nutrient in their respective omission plot, lack of the nutrient under evaluation added to the crop, and the mean soil nutrient content before soybean sowing in two climate regions were showed in Table 2, and the growing degree days (GDD) and soil average temperature during soybean growth season in two climate regions were showed in Table 3.

### Statistical analysis

Differences in average daily temperature and total precipitation during the soybean growing season, soybean seed yield, nutrient PFP, and soil indigenous nutrient supply among years or regions were analyzed using one-way analysis of variance, and their means were compared based on the least significant difference at *P* < 0.05 using the SPSS 19.0 software package for Windows (SPSS, Inc., Chicago, IL, USA). Relationships between seed yield and nutrient uptake (for N, P, and K) were implemented with the SPSS 19.0 software package for Windows using the power function (Fig. 5A,C,E). Relationships between nutrient uptake ratios (for N/P and N/K) were fit using a linear function with Microsoft Excel for Windows (Fig. 6A,C,E).
